# Sensitivity Enhancement of Pb(II) Ion Detection in Rivers Using SPR-Based Ag Metallic Layer Coated with Chitosan–Graphene Oxide Nanocomposite

**DOI:** 10.3390/s19235159

**Published:** 2019-11-25

**Authors:** Nurul Fariha Lokman, Nur Hidayah Azeman, Fatihah Suja, Norhana Arsad, Ahmad Ashrif A Bakar

**Affiliations:** 1 MyBioREC, Faculty of Civil Engineering, Universiti Teknologi MARA (UiTM), Shah Alam 40450, Selangor, Malaysia; nurul_fariha@uitm.edu.my; 2 Photonics Technology Laboratory, Centre of Advanced Electronic and Communication Engineering (PAKET), Faculty of Engineering and Built Environment, Universiti Kebangsaan Malaysia, Bangi 43600, Selangor, Malaysia; noa@ukm.edu.my; 3 Smart and Sustainable Township Research Centre (SUTRA), Faculty of Engineering and Built Environment, Universiti Kebangsaan Malaysia, Bangi 43600, Selangor, Malaysia; fati@ukm.edu.my

**Keywords:** surface plasmon resonance, silver, chitosan, graphene oxide, heavy metals, river

## Abstract

The detection of Pb(II) ions in a river using the surface plasmon resonance (SPR)-based silver (Ag) thin film technique was successfully developed. Chitosan–graphene oxide (CS-GO) was coated on top of the Ag thin film surface and acted as the active sensing layer for Pb(II) ion detection. CS-GO was synthesized and characterized, and the physicochemical properties of this material were studied prior to integration with the SPR. In X-ray photoelectron spectroscopy (XPS), the appearance of the C=O, C–O, and O–H functional groups at 531.2 eV and 532.5 eV, respectively, confirms the success of CS-GO nanocomposite synthesis. A higher surface roughness of 31.04 nm was observed under atomic force microscopy (AFM) analysis for Ag/CS-GO thin film. The enhancement in thin film roughness indicates that more adsorption sites are available for Pb(II) ion binding. The SPR performance shows a good sensor sensitivity for Ag/CS-GO with 1.38° ppm^−1^ ranging from 0.01 to 5.00 ppm of standard Pb(II) solutions. At lower concentrations, a better detection accuracy was shown by SPR using Ag/CS-GO thin film compared to Ag/CS thin film. The SPR performance using Ag/CS-GO thin film was further evaluated with real water samples collected from rivers. The results are in agreement with those of standard Pb(II) ion solution, which were obtained at incidence angles of 80.00° and 81.11° for local and foreign rivers, respectively.

## 1. Introduction

The contamination of Pb(II) in the rivers is deteriorating due to the rapid industrial development nearby the river areas [1], such as agriculture, mining activities [2], and textile industries [3]. Rivers are contaminated by Pb(II) either by the direct discharge of waste or through the leaching of Pb(II) from the soil into the rivers [3,4]. It is reported that the accumulation of Pb(II) is found in marine organisms, especially fish that live in the contaminated rivers [4]. Humans become contaminated with Pb(II) via the consumption of seafoods such as fishes, shrimps, and crabs, where Pb(II) is highly accumulated in these living organisms [5].

The hazardous nature of Pb(II) ranks it among the priority of heavy metals that are of public health concern. Pb(II) is toxic to humans even at low concentration levels where it leads to serious illnesses such as chronic inflammation of the kidney and heart, inhibited brain development, poor nerve conduction, reduction in hemoglobin formation, infertility, and abnormalities in pregnancy [6,7]. The permissible limit of Pb(II) for human consumption by the World Health Organization (WHO) is 10 ppb [8]. Furthermore, the United States Environmental Protection Agency (US EPA) has classified Pb(II) as a probable human carcinogen [2]. Since the rivers are the main water source for human consumption in most part of the world, there is an urgent need to develop a more practical technique to sense and monitor the Pb(II) content in the rivers prior to usage.

Up to now, the well-known methods in the detection of the heavy metals in the river are atomic absorption spectrometry [9], inductively coupled plasma mass spectrometry [10], anodic stripping voltammetry [11], and dynamic light scattering [12]. However, these methods generally require expensive equipment, tedious pretreatment steps, and longer analysis times [13,14]. The existing techniques can be improved by introducing the optical sensing technique for Pb(II) ion detection in water with the advantages of rapid, sensitive selectivity toward the target analyte; one such technique is surface plasmon resonance (SPR) sensor. SPR is one of the excellent optical sensor techniques for heavy metals detection due to their apparent advantages over the conventional methods such as an inexpensive, reliable procedure, and high sensitivity toward heavy metal ions [15]. The SPR is an optical process in which p-polarized light causes the excitation of a charge density wave along the metal–dielectric material interface by satisfying a resonance condition [16]. In other words, at a certain incident angle, when the wave vector of incident light matches the wave vector of the surface plasmon wave, a sharp dip is produced in the reflectivity curve [17]. A thin metallic layer such as a gold (Au) or silver (Ag) layer is usually used to excite the surface plasmon wave on the SPR surface.

Selection of the metallic layer is important for producing higher sensor sensitivity. The application of Au thin layer film is commonly used due to its superior stability compared to Ag [18]. Ag suffers from oxidation and sulfuration, which limits some of its SPR performance. However, a recent study shows that Ag demonstrates better field enhancement and penetration depth in comparison to Au by 5 and 1.4 times, respectively [19], resulting in the better sensitivity of the sensors. In another report, Homola and co-workers [20] demonstrated that the propagation length for Ag is approximately twice that of Au in near-infrared region wavelengths, which shows its intrinsic sensitivity enhancement over Au. Therefore, to overcome the limitation of using Ag, the application of coatings using other materials on top of the Ag surface such as polymers [21] and carbon-based [22] materials is essential, where it acts as the protecting layer for Ag.

Chitosan (CS) is a type of natural-based polymer that is known for its high affinity toward heavy metal ions such as Pb(II) [23]. The active functional groups on the CS surface such as amino (–NH_2_) and hydroxyl (–OH) functional groups play an important role in sensing Pb(II) ions. The first report on the application of CS as the sensing material for Pb(II) ions in an SPR sensor was published by Fen et al. (2013) [24] with 0.5 ppm as the lowest detection limit. Later in 2018, Abdullah et al. [25] used CS as the active sensing layer for Pb(II) ion sensing using the localized SPR sensors with the sensitivity of 0.8165 nm ppm^−1^. However, the utilization of CS as the standalone-based material creates low physicochemical and mechanical properties for the sensor’s application. Therefore, glutaraldehyde, which acts as the crosslinking agent, is added to improve the chemical stabilities of CS [24]. To further improve the properties of the active sensing layer, graphene-based material is added into the CS solution.

Graphene is a single two-dimensional (2D) plane of carbon atoms forming a hexagonal lattice structure that confines stronger surface plasmon waves than other metallic layers [26]. However, the lack of functional groups on the surface of graphene limits its performance. Therefore, modifying the graphene structure through an oxidation process is necessary, where many functional groups such as hydroxyl (–OH), epoxy (–O), and carboxyl (–COOH) are added in the graphene structure [23]. These functional groups influence a large specific surface area, which is excellent for sensing application and is able to chelate with heavy metal ion as well [25]. Reports on the SPR performance for Pb(II) ion detection is common; however, to the best of our knowledge, this is the first report on SPR performance based on an Ag metallic layer integrated with a chitosan–graphene oxide (CS-GO) nanocomposite thin film for Pb(II) ion detection using contaminated river water samples. Herein, we report on the proof of concept for Pb(II) ion sensing using an SPR-based Ag metallic layer integrated with a CS-GO nanocomposite thin film and the application of the developed SPR sensors on real water samples collected from the contaminated rivers to validate the SPR performance. The CS-GO nanocomposite thin layer film acts as the protective layer for Ag from oxidation and sulfuration. The sensor performance in terms of the sensitivity and detection accuracy of the prepared thin film for Pb(II) ion detection using the SPR sensor technique is also evaluated.

## 2. Materials and Methods

### 2.1. Materials

Chitosan (75–85% deacetylated), graphene oxide (GO), acetic acid, glutaraldehyde (GLA), Pb(II) ion standard solution, and Pb(II) ionophore were all obtained from Aldrich. All chemicals are of analytical grade.

### 2.2. Preparation of the Sensing Material

Three different solutions were prepared for the sensing materials. For solution I, 0.4 g of chitosan was dissolved in 50 mL of 1% acetic acid followed by 0.05 g of glutaraldehyde. Then, the solution was stirred for 1 h. Meanwhile, to prepare solution II, the same procedure was repeated as in solution I preparation but with an additional of 3 mL of GO in the solution. The GO concentration used was 1 mg/mL. Then, the mixture was stirred for 1 h and sonicated for 10 min at 53 °C. Then, solution III was prepared by repeating the procedure for the preparation of solution II but with an additional of 5 mL of ionophore. Then, the mixture was stirred for 1 h and sonicated for 10 min at 53 °C. Ionophore was added to enhance the selectivity of the SPR sensors toward the Pb(II) ions. The prepared solutions were characterized using an X-ray photoelectron spectroscopy (XPS) model Kratos AXIS Ultra Delay-Line Detector (DLD) to observe the chemical composition of the material. Meanwhile, the physicochemical properties and surface morphology of the material were carried out using an X-ray diffraction (XRD) model Siemens D-5000 and atomic force microscopy (AFM) model NTEGRA Prima.

### 2.3. Fabrication of Nanostructure Thin Films

Three samples of Ag/CS, Ag/CS-GO, and Ag/CS-GO-Ionophore nanostructure thin films were prepared on top of the glass substrates, separately. The glass substrates, which have a 24 mm × 24 mm dimension area and 0.13–0.16 mm thickness range, were obtained from Menzel-Glaser. The glass substrates were cleaned using acetone to ensure that the surface was free from any impurities. A thin silver film (~50 nm) was deposited on top of the substrates using a PVD 75 Sputter Coater controlled by a film thickness monitor. The substrates were located at 35 mm from the silver foil target (99.99% in purity) and deposited at a rotational speed of 10 rpm for 360 s. The sputter deposition was carried out with a power of 50 W under an argon pressure of approximately 5 × 10^−3^ Torr at room temperature.

**Figure 1:**
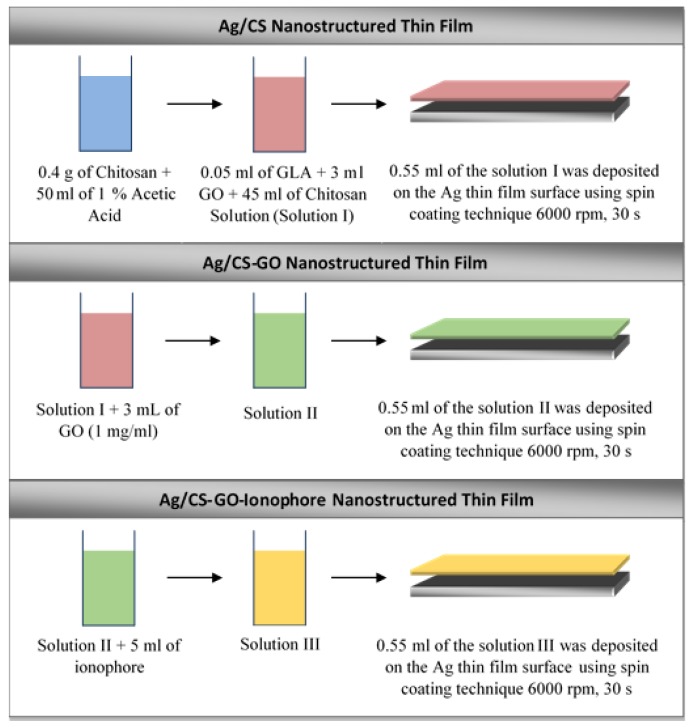
Schematic procedure of fabrication process for Ag/chitosan–graphene oxide (CS-GO) nanostructure thin films.

**Figure 2:**
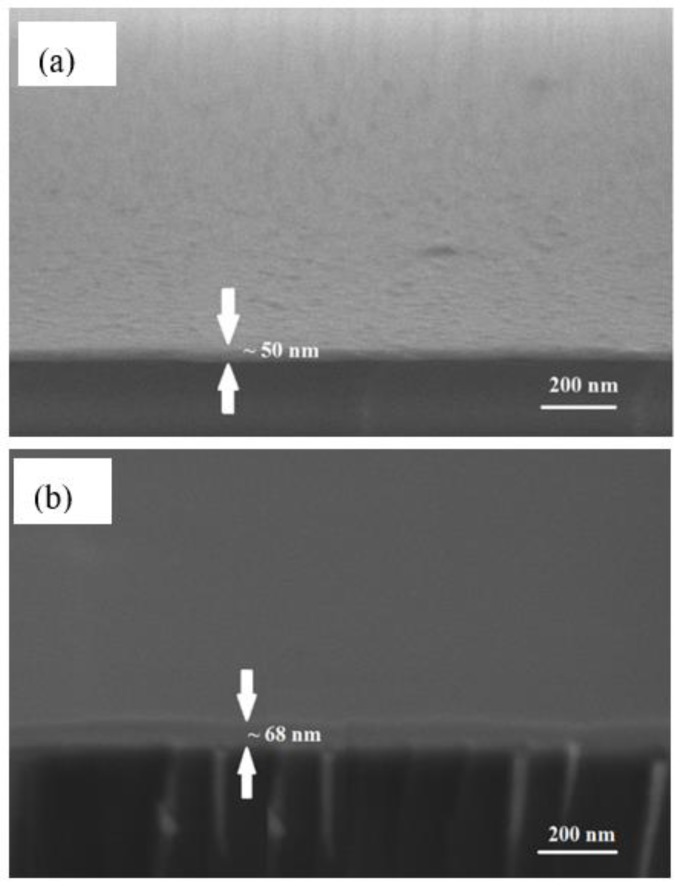
FESEM images of the Ag thin films thickness on the surface of the glass substrate (**a**) before and (**b**) after being coated with the sensing materials layer.

Next, approximately 0.55 mL of solution I, II, and III was coated on the surface of the silver thin film substrates, separately, using a Laurell Technologies WS-400BX-6NPP spin coater. The spin coater was operated at 6000 rev/min for 30 s. Figure 1 shows the schematic procedure of the fabrication process for Ag/CS, Ag/CS-GO, and Ag/CS-GO-Ionophore nanostructure thin films. Meanwhile, Figure 2 shows the field emission scanning electron microscope (FESEM) images of the Ag thin film’s thickness on the surface of the glass substrate before and after being coated with the sensing material layer. The FESEM image in Figure 2a confirms that the thickness of Ag on the glass substrate is 50 nm, where it is reported that the SPR sensor performance is optimum at this metal thickness [19]. After the addition of the sensing material film, the thickness of the nanostructure thin film increases by approximately ~68 nm, as shown in Figure 2b. The thickness of the sensing materials is approximately similar for all the three solutions used.

### 2.4. Preparation of the Samples

#### 2.4.1. Standard Pb(II) Solutions

Working standard solutions of Pb(II) were prepared by diluting 1000 ppm standard solution with deionized water to six different concentrations (0.03 ppm, 0.10 ppm, 0.50 ppm, 1.00 ppm, 3.00 ppm, and 5.00 ppm).

#### 2.4.2. Real Water Sample

The real water samples were collected from local and foreign rivers. The local sample was collected from Klang river, Malaysia, while the foreign sample was collected from Citarum river, Indonesia. Figure S1a,b shows the sampling sites of the Klang and Citarum rivers, respectively. The Klang and Citarum rivers were selected in this study due to their high heavy metal content, particularly Pb(II) ions in these two rivers [1,2]. Approximately 200 mL of river water samples were collected using the grab sampling technique and kept in a polyethylene container. Then, the water samples were placed in a polystyrene box which was tightly sealed, and the temperature was kept constant at 4 °C during the transportation to the laboratory. Upon arrival to the laboratory, the samples were transferred into the refrigerator and stored at 4 °C until further use [27].

### 2.5. SPR Response

**Figure 3:**
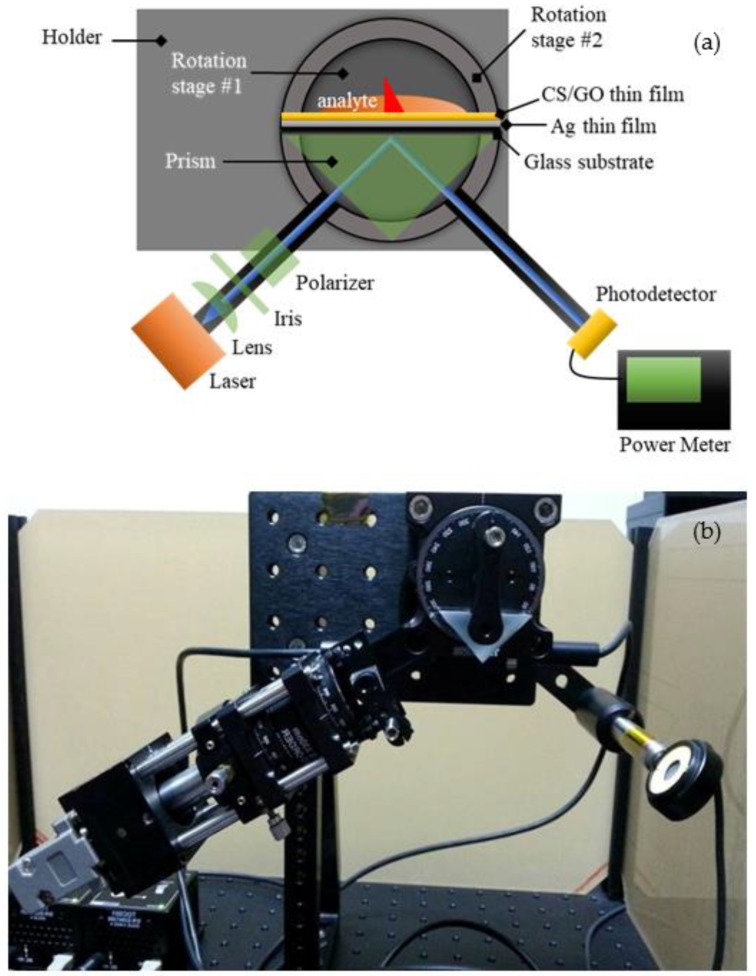
(**a**) Schematic diagram and (**b**) real surface plasmon resonance (SPR) sensor system setup.

SPR reflectance were measured using an SPR setup that was equipped with an 850 nm laser light source, as shown in Figure 3. The laser light source is incident on a metal film in contact with a prism, and its reflectance is detected with a photodetector. The setup consists of a prism, two rotation stages, a laser, a lens, an iris, a polarizer, a photodetector, and an optical power meter. The nanostructure thin film samples were attached to the uncoated right-angle prism (N-BK7, Thorlabs, Newton, NJ, USA) using a refractive-index-matching gel (glycerine). An analyte, such as a Pb(II) ion solution, was placed on top of the sample. The prism was positioned separately on top of a motorized rotation stage with two worm drives (CR1/M-Z7E, Thorlabs). The rotation stage was utilized to rotate the laser light and the photodetector (S120VC, Thorlabs) corresponding to the measured angle. An 850 nm wavelength vertical-cavity surface-emitting laser (VCSEL, Thorlabs) with 1 mW output power was employed to transmit light into the right-angle prism. The transmitted light was coupled using an aspheric lens (C240TME-B, Thorlabs), which was then optimized with an iris (SM1D12, Thorlabs) and polarized using a polarized beam splitter (PBS202, Thorlabs), such that only p-polarized light passed through the prism and made contact with both the thin film and the analyte. The change in the reflected light corresponds to the SPR reflectance of the nanostructure thin films interacting with the analytes, and it was detected using the photodetector and measured using the optical power meter (PM100D, Thorlabs). Pure deionized water and different concentrations of Pb(II) are tested as analytes on top of the Ag/CS, Ag/CS-GO, and Ag/CS-GO-Ionophore nanostructure thin films. The SPR reflectance of different analytes is presented as an SPR curve, which represents the sensitivity of the SPR sensor.

## 3. Results and Discussions

### 3.1. Characterization of the Material

**Figure 4:**
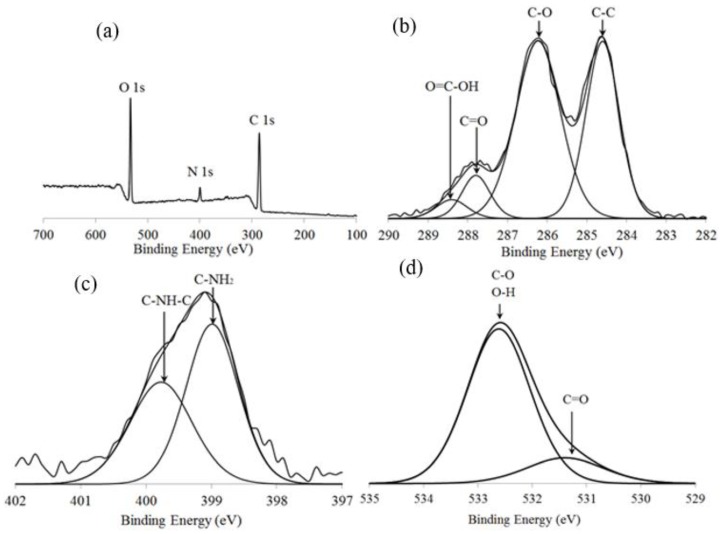
X-ray photoelectron spectroscopy (XPS) spectra for the CS-GO thin film of the (**a**) wide scan spectra, (**b**) narrow scan of C 1s peak, (**c**) narrow scan of N 1s spectra, and (**d**) narrow scan of O 1s spectra.

Figure 4 shows the XPS analysis for the CS-GO nanocomposite thin film. Figure 4a shows the overall chemical compositions that are present in the CS-GO thin film, which consists of O 1s, N 1s, and C 1s elements. It was observed that the O 1s element was the highest amount of element present in the CS-GO thin film, which was contributed by both chitosan and graphene oxide. The high amount of O 1s element in the CS-GO thin film provides many active sites for the adsorption of Pb(II) ions [28]. In Figure 4b, the C–C binding at the 284.6 eV peak corresponds to the sp^2^ hybridized. Meanwhile, the C–O (286.2 eV) and C=O (287.8 eV) correspond to the carbonyl functional group, and the O=C–OH (288.5 eV) corresponds to the carboxyl functional group [29]. The N 1s spectra in Figure 4c shows that C–NH_2_ (398.9 eV) and C–NH–C (399.8 eV) are attributed to the amine and amide functional groups in the chitosan structure, respectively [30]. The presence of C=O (531.2 eV), C–O, and O–H (532.5 eV) functional groups in Figure 4d confirms the successful synthesis of the CS-GO nanocomposite [31].

### 3.2. Physical Properties of the Sensing Material

**Figure 5:**
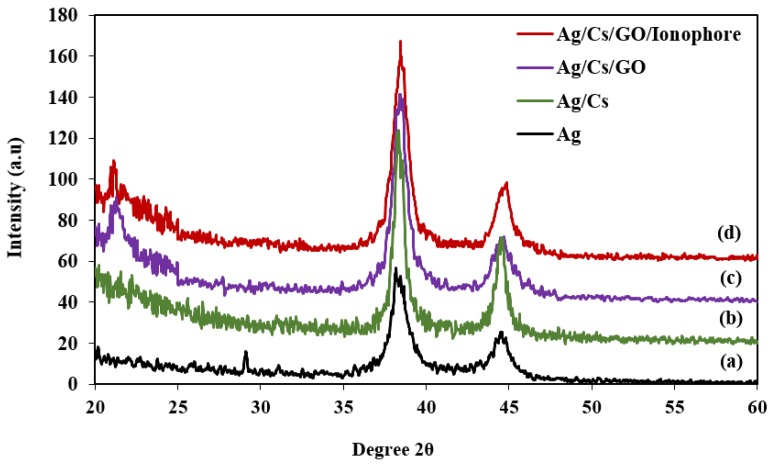
X-ray diffraction (XRD) spectra for (**a**) Ag, (**b**) Ag/CS, (**c**) Ag/CS-GO, and (**d**) Ag/CS-GO-Ionophore nanostructure thin films on the surface of the glass substrate.

XRD was carried out to study the crystallinity and amorphous properties of the materials. Figure 5 shows the XRD spectra for (a) Ag, (b) Ag/CS, (c) Ag/CS-GO, and (d) Ag/CS-GO-Ionophore nanostructure thin films on the surface of the glass substrate. Figure 5a shows the appearance of the two peaks at 38.2° and 44.5°, which correspond to the (111) and (200) reflections, respectively, hence confirming that the Ag is coated on the surface of the glass substrate [32]. The result matched with the Joint Committee on Powder Diffraction Standards (JCPDS No. 00-004-0783), which are ascribed to the Ag, and the crystal structure was identified as the face-centered cubic (fcc). The crystallite size was calculated to be 77.61 nm.

After the CS thin film is coated on the surface of Ag (Figure 5b), a broad peak in the 2θ range from 20.0° to 30.0° is observed, which confirms the presence of CS on the Ag/CS nanostructure thin film [33]. Furthermore, the broad peak observed at 2θ ranging from 23.0° to 28.0° indicates the amorphous properties and the presence of a polymeric chain in CS [33]. Meanwhile, two diffraction peaks are observed at 38.3° and 44.5°, which correspond to the (111) and (200) planes of the monoclinic, respectively. This result is in good agreement with the JCPDS value (00-038-0786), which are attributed to Ag/CS. The crystallite size of the Ag/CS thin film is 122.27 nm, where a slight improvement in crystallinity is observed compared to that of the Ag thin film. A slight increase in the crystallite size for Ag/CS is due to the Ag crystalline structure being stabilized by the polymeric matrix [34].

Due to the amorphous structure of CS not being affected by the addition of GO, a similar trend is observed for the Ag/CS-GO thin film in Figure 5c and that of the Ag/CS thin film. The prominent peaks for the Ag/CS-GO thin film that were observed at 2θ = 38.4° and 44.6° correspond to the (111) and (200) planes, respectively. A similar finding is reported by Mao et al. (2012) [35], who stated that the high crystallinity of Ag dominates the appearance of carbon in the GO sheets. The addition of GO in the CS produces a smaller crystallite size of about 90.1 nm, which is smaller than that of Ag/CS [36]. This is due to the viscosity properties of GO, which slows the growth of the crystallite size in CS. It is suggested that a smaller crystallite size provides a larger surface area for the interaction of sensing material with the chemical analyte.

Ionophore is added to enhance the selectivity of the SPR sensors toward the Pb(II) ions, and it is not involved in the chemical reaction with the thin film produced. Therefore, a similar XRD pattern is observed for Ag/CS-GO-Ionophore, as seen in Figure 5d, as with that of Ag/CS and Ag/CS-GO thin films. The diffraction peaks appear at 2θ = 38.4° and 44.6°, which correspond to the (111) and (200) planes, respectively. The crystallite size is calculated to be approximately 93.71 nm.

### 3.3. Surface Morphology of the Nanostructure Thin Film

**Figure 6:**
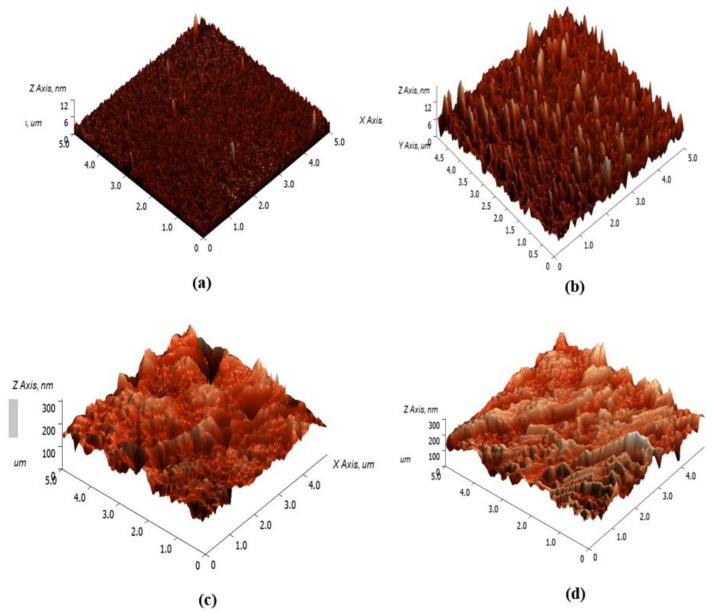
Atomic force microscopy (AFM) images of the (**a**) Ag, (**b**) Ag/CS, (**c**) Ag/CS-GO, and (**d**) Ag/CS-GO-Ionophore thin films.

Figure 6 shows the AFM images of the (a) Ag, (b) Ag/CS, (c) Ag/CS-GO, and (d) Ag/CS-GO-Ionophore thin films. In Figure 6a, Ag is distributed uniformly on the glass substrate, and a smooth surface was produced with the root mean square (RMS) roughness of 1.418 nm. When CS was added on the Ag surface (Figure 6b), the Ag structure was not entirely visible because of masking by the CS layer [37]. However, the surface roughness slightly increases to 1.647 nm due to the presence of the functional group on the chitosan structure [25]. The incorporation of GO increases the surface roughness of Ag/CS-GO up to 17 times compared to that of the Ag/CS thin film due to the agglomeration of GO, as shown in Figure 6c. Meanwhile, Figure 6d exhibits that the surface structure of Ag/CS-GO-Ionophore is almost similar to that of Ag/CS-GO with the surface roughness of 31.040 nm.

### 3.4. Sensing Mechanism

**Figure 7:**
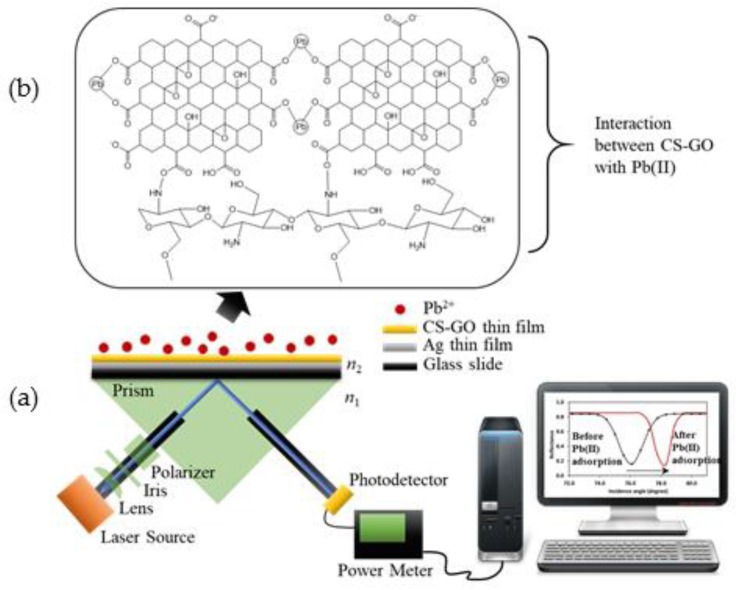
(**a**) Proposed sensing mechanism for the detection of Pb(II) using CS-GO as an active layer for surface plasmon resonance (SPR) sensors and (**b**) the chemical interaction between CS-GO with Pb(II).

In this work, the sensing mechanism is based on the interaction of an evanescent wave with the chemical analytes on the surface of the SPR prism. In Figure 7a, the light that enters the prism at an angle larger than that of critical angle undergoes total internal reflections due to the propagation of light from higher (n_1_) to lower refractive index medium (n_2_). During this occurrence, there is an amount of the incident light that exists at the interface of both media, which is called an evanescent wave [38]. When an Ag thin film is placed in between the two different refractive index media, the evanescent wave is enhanced beyond the n_2_ surface region [39], leading to sensitivity enhancement in SPR. The CS-GO thin film that is coated on the Ag thin film surface acts as the active sensing layer for Pb(II) ion detection. The interaction of light with Ag/CS-GO before and after Pb(II) ion adsorption produces a shift in the SPR angle due to a change in the refractive index. Figure 7b shows the chemical structure of the CS-GO composite and the possible electrostatic interaction that occurs between the CS-GO composite with the Pb(II) ions.

### 3.5. SPR Responses

#### 3.5.1. SPR Sensitivity

**Figure 8:**
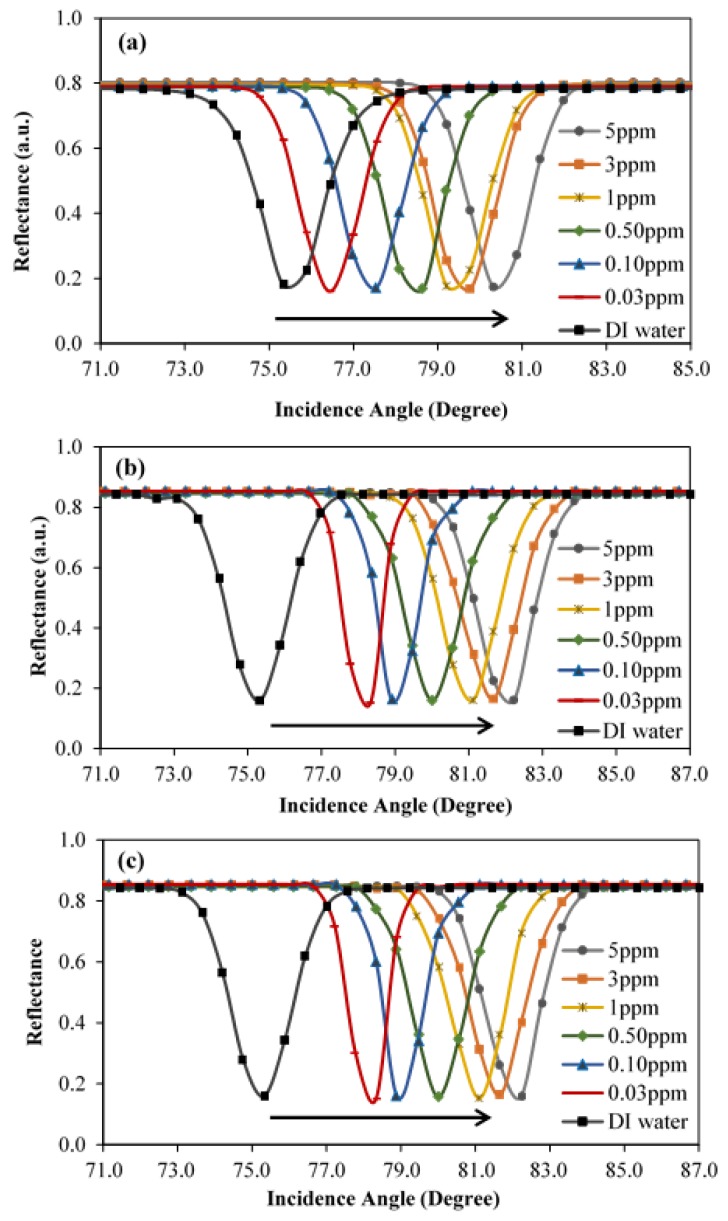
The SPR curves for (**a**) Ag/CS, (**b**) Ag/CS-GO, and (**c**) Ag/CS-GO-Ionophore nanostructure thin films toward deionized water and different concentrations of Pb(II).

The SPR curves for the Ag/CS, Ag/CS-GO, and Ag/CS-GO-Ionophore thin films toward pure deionized water and different concentrations of Pb(II) are presented in Figure 8a–c, respectively. Pure deionized water and six different concentrations of Pb(II) in the range of 0.03 to 5.00 ppm were prepared. The initial incidence angle was measured when the thin film made contact with the pure deionized water, and a new incidence angle appeared with different concentrations of the analyte. The incidence angles occurred at the minimum reflectance and were illustrated by the SPR curve. Taking the initial incidence angle as a baseline and comparing to the new incidence angle, the difference in the incidence angle (∆θ) over the concentration of Pb(II) will be calculated as the sensitivity of the SPR sensors.

The incidence angle trend in Figure 8a was measured to be shifted to the right when the Ag/CS thin film makes contact with higher concentrations of the Pb(II). The initial incidence angle is 75.32° where minimum reflectance occurred for pure deionized water. The new incidence angle for 0.03 ppm Pb(II) increases to 76.43° and is equivalent to ∆θ of 1.11°. We observed that the increment in the incidence angle is proportional to the increase in concentration of Pb(II). Thus, the ∆θ values for 0.10 ppm, 0.50 ppm, 1 ppm, 3 ppm, and 5 ppm of Pb(II) ions were measured and increased to 2.22°, 3.33°, 3.89°, 4.44°, and 5.00°, respectively. The sensitivity of SPR for the Ag/CS thin film toward 5 ppm of Pb(II) was found to be higher, corresponding to 1.00° ppm^−1^ compared to the work reported in reference [40] with 0.00483° ppm^−1^. The large difference was attributed to the use of Ag as a metallic layer where the penetration depth into dielectric at an 850 nm wavelength was reported higher in [20] compared to Au.

Figure 8b shows the SPR curves for the Ag/CS-GO thin films toward deionized water and different Pb(II) concentrations. Although the incidence angle trend seems similar to that in Figure 8a, the ∆θ value in Figure 8b is more distinct, which reveals the potential of the Ag/CS-GO thin films to measure a wide concentration range of heavy metal. Upon deionized water exposure, the incidence angle occurred at the minimum reflectance of 75.32°. When the thin film makes contact with 0.03 ppm of Pb(II), the incidence angle that accounted for 78.33° corresponded to a ∆θ of 3.01°. In addition, as the Pb(II) concentrations increase to 0.10 ppm, 0.50 ppm, 1 ppm, 3 ppm, and 5 ppm, the ∆θ values were found to increase accordingly to 3.57°, 4.68°, 5.79°, 6.35°, and 6.90°, respectively. With the inclusion of GO, the SPR sensitivity for the Ag/CS-GO was enhanced to 1.38° ppm^−1^ compared to the sensitivity of the Ag/CS thin film. Reports point out that a sharper resonance curve means that a better resolution may enhance the surface plasmon resonance, and thus it increases the sensitivity [17]. This statement agreed well with the sharper resonance curve in Figure 8b compared to Figure 8a. Furthermore, with the inclusion of GO, more additional binding sites provided by GO are available for Pb(II) ion binding, hence leading to an increase in sensitivity [16,18].

Meanwhile, Figure 8c shows the SPR curves for the Ag/CS-GO-Ionophore thin film using the same range concentrations of Pb(II). The SPR spectra of Ag/CS-GO-Ionophore shows no significant difference with the Ag/CS-GO before the addition of ionophore, as depicted in Figure 8b. The same magnitude of ∆θ was observed for all Pb(II) ion concentrations used; therefore, the SPR sensitivity remained unchanged at 1.38° ppm^−1^. It is suggested that the unchanged sensitivity of the SPR sensor when using the Ag/CS-GO-Ionophore nanostructure thin film for the detection of Pb(II) is due to the high density of GO.

Based on this result, it is suggested that the addition of GO greatly enhanced the sensitivity of the SPR sensor without the use of ionophore. Previously, it is reported that Ag thin films in SPR are unstable and easily become oxidized when used without a protective layer, hence affecting the sensor performance. However, in this work, a good sensitivity was obtained, which was mainly due to the application of CS-GO as the sensing layer for Pb(II) ions, since at the same time, it plays an important role as the protective layer for Ag.

**Figure 9:**
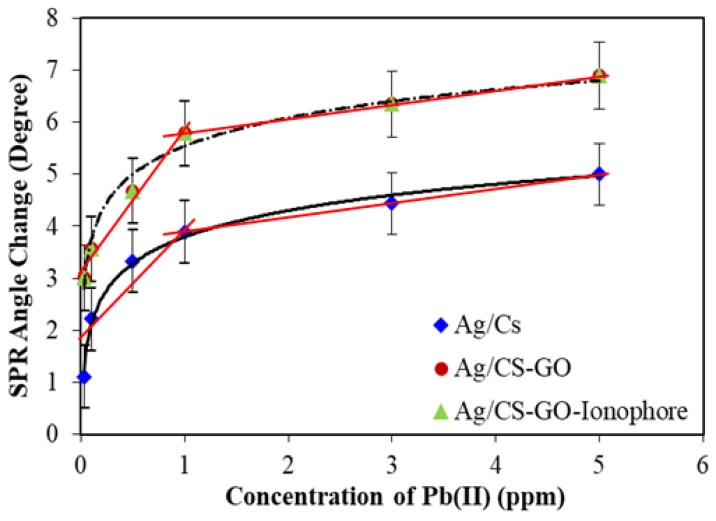
The SPR calibration curve for Ag/CS, Ag/CS-GO, and Ag/CS-GO-Ionophore nanostructure thin films toward deionized water and different concentrations of Pb(II) ions.

Figure 9 shows that the plots of resonance angles change (∆θ) as a function of Pb(II) concentration ranging from 0.03 to 5 ppm for Ag/CS, Ag/CS-GO, and Ag/CS-GO-Ionophore nanostructure thin films. The graph shows that the ∆θ increases proportionally with the increase of Pb(II) concentrations for all the three nanostructure thin films. However, the graph started to reach saturation after 1 ppm of Pb(II) concentration, suggesting that the binding site on the surface of the sensing materials is fully occupied [25]. As discussed in the previous section, Ag/CS-GO and Ag/CS-GO-Ionophore show similar sensitivity, which was better than that of the Ag/CS thin film. Therefore, a similar R-squared value was observed for Ag/CS-GO and Ag/CS-GO-Ionophore with R^2^ = 0.9767. The value is slightly higher than that of Ag/CS (R^2^ = 0.8401) with a 14% difference. It is suggested that the addition of GO improves the sensitivity of the SPR sensor due to the additional binding site provided by GO; hence, higher sensitivity was obtained for Ag/CS-GO and Ag/CS-GO-Ionophore in comparison to the Ag/CS nanostructure thin film. It is also suggested that the increase in SPR sensitivity using Ag/CS-GO and Ag/CS-GO-Ionophore thin films in comparison to that of Ag/CS is due to the higher surface roughness that was observed for Ag/CS-GO and Ag/CS-GO-Ionophore thin films, which was proven by AFM analysis. The higher surface roughness of the thin film indicated a higher surface to volume ratio for Pb(II) ion binding [18].

#### 3.5.2. Detection Accuracy

**Figure 10:**
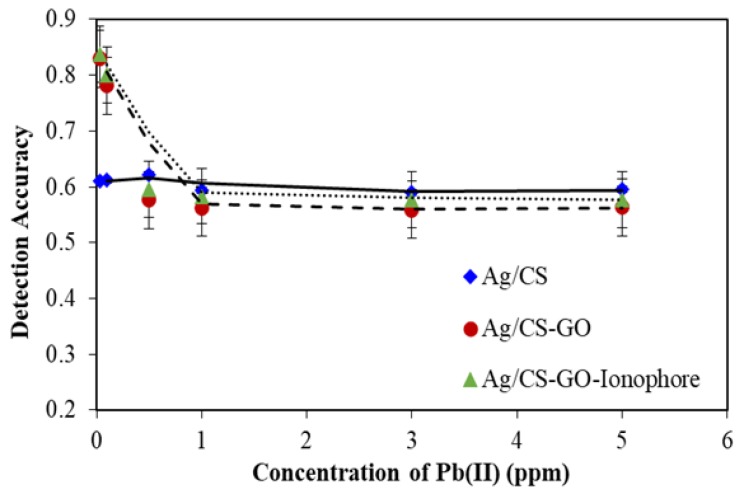
The detection accuracy for Ag/CS, Ag/CS-GO, and Ag/CS-GO-Ionophore nanostructure thin films toward different concentrations of Pb(II) ions.

Figure 10 shows the detection accuracy as a function of Pb(II) concentration from 0.03 to 5 ppm for Ag/CS, Ag/CS-GO and Ag/CS-GO-Ionophore nanostructure thin films. The detection accuracy is the reciprocal of the SPR curve full-width-half-maximum (θ_½_), which can be estimated from the following equation, as defined in [38].

Detection accuracy = (1/∆θ_½_)

where ∆θ_½_ is the width of the SPR curve at the level of reflectance corresponding to half of its maximum value.

The detection accuracy depends on the width of the SPR curve, where the narrower SPR curve represents the higher detection accuracy [23]. At lower concentration, the detection accuracy for Ag/CS-GO and Ag/CS-GO-Ionophore thin films are higher in comparison to that of Ag/CS film, showing a better performance for the SPR sensor. This corresponds to the discussion in Figure 8 where a narrower SPR curve has been observed for Ag/CS-GO and Ag/CS-GO-Ionophore thin films. However, the graph trend seems to be stagnant at higher concentrations starting from 1 ppm of Pb(II) and beyond. It is suggested that the greater binding of Pb(II) ions on the surface of the nanostructure thin films contributed to this event. Thus, this suggests that the better performance of the proposed sensor can be tuned by tuning the film used and the range of required Pb(II) ion concentration.

#### 3.5.3. Selectivity of the Sensors

**Figure 11:**
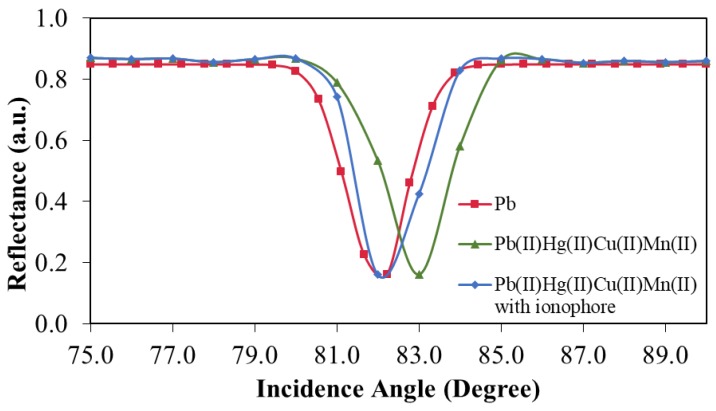
Selectivity study for the Ag/CS-GO nanostructure thin film when exposed to solutions containing Pb(II) ions and a mixture of Pb(II), Hg(II), Cu(II), and Mn(II).

Figure 11 shows the selectivity of the Ag/CS-GO nanostructure thin film for Pb(II) ion detection at a concentration of 1 ppm. In this work, the pH values for all the metal solutions were controlled in the range of 6.0 to 6.5 to maintain good SPR response. When Ag/CS-GO was exposed to the single Pb(II) metal ion solution, the degree of incidence angle is at 82.22°. When the Ag/CS-GO thin film was exposed to the mixture of metal ion solution containing Pb(II), Hg(II), Cu(II), and Mn(II), the angle of incidence slightly shifted to the right at 83°. The slight shift in angle is probably due to the presence of interference in the solution. However, when ionophore was added into the solution, the angle of incidence was similar to that of the single Pb(II) metal ion solution which is at 82°, hence showing that the sensor selectivity is enhanced through the addition of ionophore and shows good selectivity toward Pb(II) ions.

### 3.6. Analysis on Real Water Sample

The sensor performance was further evaluated with an analysis on a real water sample. Real water samples were collected from local and foreign rivers such as the Klang and Citarum rivers, respectively, as these rivers are highly contaminated with heavy metals, particularly Pb(II) ions.

The water samples were collected from five (5) different points from each river and were first analyzed using an established method for heavy metals analysis in waters, which is known as inductively coupled plasma–atomic emission spectrometry (ICP-AES). The results were compared with the standard guidelines to confirm the contamination status of these rivers. The average experimental results shows that the Pb(II) concentration in the Klang river was 0.40 ppm with standard deviation and error value of 0.019 and 0.004%, respectively. Meanwhile, the average Pb(II) concentration in the Citarum river was 0.98 ppm with the standard deviation and error value of 0.011 and 0.002%, respectively. Both of the rivers are exceeding the permissible limit recommended by the National Water Quality Standard (NWQS) with 0.05 ppm. The Pb(II) concentration value obtained by ICP-AES analysis from each river was used as a guideline to validate the SPR sensors technique. The results of the Pb(II) average concentration analyzed using the ICP-AES method is shown in Figure S2.

**Figure 12:**
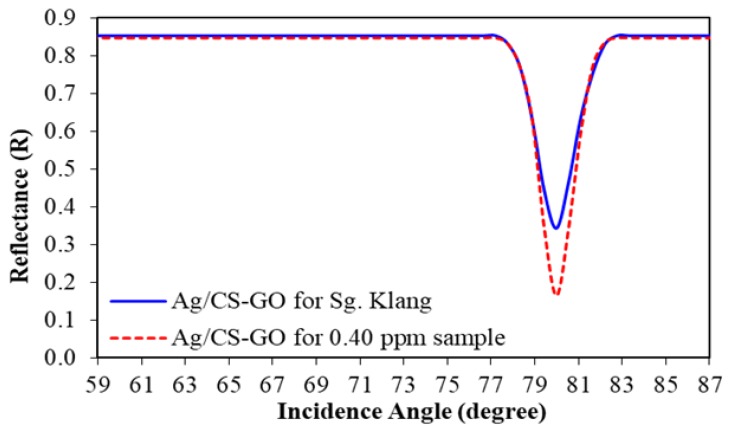
The SPR curve for Ag/CS-GO nanostructure thin film of the Klang river.

To study the SPR response for Pb(II) ion detection in the river, two types of samples were prepared: standard Pb(II) ion solution and real water samples collected from the rivers. According to the SPR analysis in Figure 9, SPR shows better sensor response when using Ag/CS-GO and Ag/CS-GO-Ionophore thin films for the detection of Pb(II) compared to that of an Ag/CS thin film. However, Ag/CS-GO and Ag/CS-GO-Ionophore show almost similar performances toward Pb(II) ion detection. Therefore, in this section, only an Ag/CS-GO thin film was used throughout the study. Figure 12 shows the SPR curve of Pb(II) ion detection using an Ag/CS-GO thin film layer for standard Pb(II) ion solution at 0.40 ppm and real water samples collected from the Klang river. The graph shows that the incidence angle is at 80.00° for the Ag/CS-GO thin film when exposed to the 0.40 ppm standard Pb(II) solution and real water sample, which is closely matched with the SPR curves in the previous section for the concentration ranging from 0.1 to 0.5 ppm (Figure 8b). The reflectance value obtained for the standard Pb(II) ion solution and real water samples are 0.1635 and 0.3420, respectively with the percentage difference of 52.19%.

**Figure 13:**
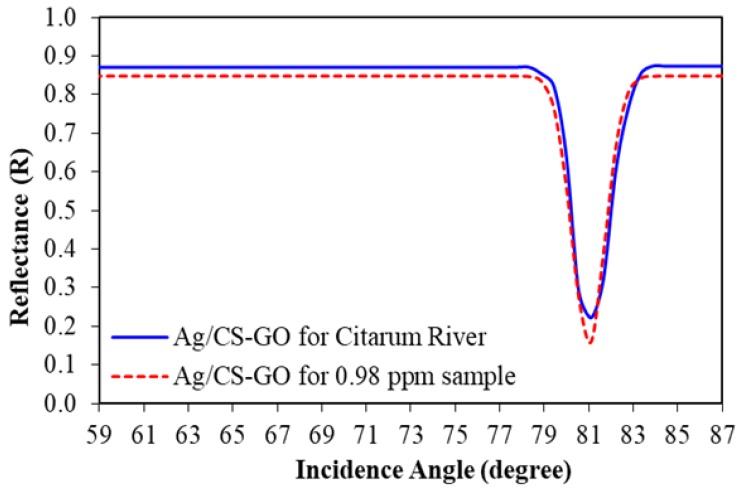
The SPR curve for Ag/CS-GO nanostructure thin film of Citarum river.

Meanwhile, Figure 13 shows the SPR curve of Pb(II) ion detection using an Ag/CS-GO thin film layer for an 0.98 ppm standard Pb(II) solution and a real water sample collected from the Citarum river. The incidence angle is observed to appear at 81.111° for the Ag/CS-GO thin film when exposed to the 0.98 ppm concentration, which is in agreement with the SPR curve in the previous section for 1 ppm concentration (Figure 8b). The reflectance was calculated to be 0.163 and 0.271 for standard Pb(II) ion solution and real water samples, respectively, with a percentage difference of 39.78%. A smaller dip was observed for both SPR curves using a real water sample compared to that of standard Pb(II) ion solution due to the presence of interference in the real water sample, which affected the SPR response [41]. From these observations, it can be concluded that the SPR technique based on Ag/CS-GO nanostructure thin film is reliable for Pb(II) ion detection in real water samples.

## 4. Conclusions

In this work, SPR-based Ag/CS-GO nanostructure thin film was successfully developed for Pb(II) ion detection using Pb(II) standard solution as well as real water samples collected from rivers. The incorporation of GO with CS increases the surface roughness of the sensing material as shown by AFM analysis, therefore providing more active sites for Pb(II) ion detection, and leading to a better sensitivity of 1.38° ppm^−1^ in comparison to Ag/CS nanostructure thin film. Furthermore, SPR based Ag/CS-GO nanostructure thin film shows better detection accuracy at lower Pb(II) concentrations, with 0.84 in comparison to Ag/CS nanostructure thin film with 0.61, which was due to the incorporation of GO with the sensing material. The evaluation of SPR performance on real water samples collected from the Klang and Citarum rivers shows nearly similar SPR performance when using standard Pb(II) solution.

## Supplementary Materials

The following are available online at https://www.mdpi.com/1424-8220/19/23/5159/s1, Figure S1: The sampling site of (a) Klang and (b) Citarum rivers in Malaysia and Indonesia, respectively, Figure S2: The average concentration of Pb(II) from Klang and Citarum rivers analyzed using ICP-AES method.
