# Supplementary material for: Sensitivity Enhancement of Pb(II) Ion Detection in Rivers Using SPR-Based Ag Metallic Layer Coated with Chitosan–Graphene Oxide Nanocomposite
Source: Sensors (Basel). 2019 Nov 25;19(23):5159. doi: 10.3390/s19235159 (PMC6928891; doi:10.3390/s19235159)
Supplement: Supplementary file 1 [file sensors-19-05159-s001.pdf]

SUPPLEMENTARY INFORMATION

Sensitivity enhancement of Pb(II) ion detection in rivers using SPR based Ag metallic layer coated with chitosan-graphene oxide nanocomposite

Nurul Fariha Lokman, [a] **Nur Hidayah Azeman,** [b,*] **Fatihah Suja,** [c] **Norhana Arsad,** [b] **Ahmad Ashrif A Bakar,** [b,§]

[a] myBioREC, Faculty of Civil Engineering, Universiti Teknologi MARA (UiTM), 40450 Shah Alam, Selangor, Malaysia.

[b] Photonics Technology Laboratory, Centre of Advanced Electronic and Communication Engineering (PAKET), Faculty of Engineering and Built Environment, Universiti Kebangsaan Malaysia, 43600 Bangi, Selangor, Malaysia.

[c] Smart and Sustainable Township Research Centre (SUTRA), Faculty of Engineering and Built Environment, Universiti Kebangsaan Malaysia, 43600 Bangi, Selangor, Malaysia.

Corresponding authors: nhidayah.az@ukm.edu.my *****; ashrif@ukm.edu.my [§]

APPENDIX A

Sampling site for real water sample

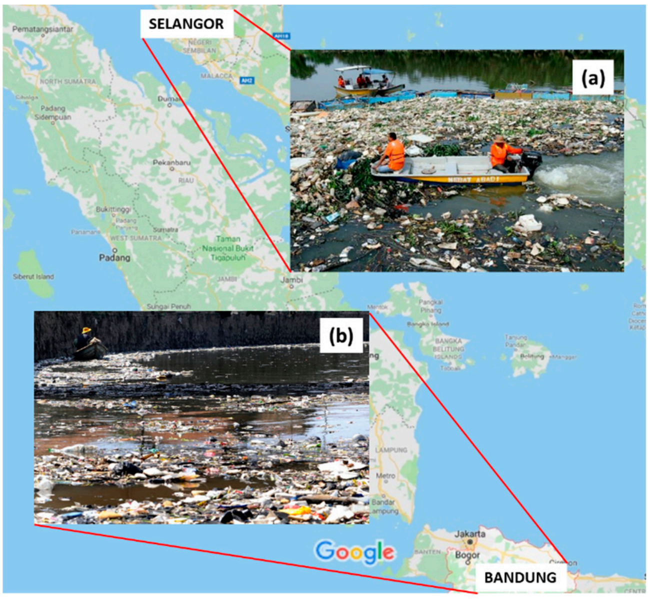

Figure S1: The sampling site of (a) Klang and (b) Citarum rivers in Malaysia and Indonesia, respectively [1]

APPENDIX B

Pb(II) Measurement using ICP-AES Method

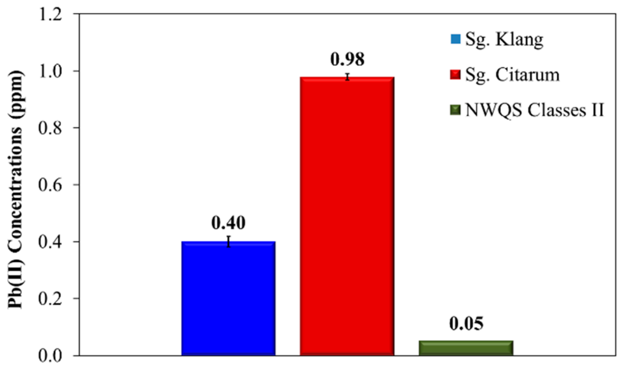

Figure S2: The average concentration of Pb(II) from Klang and Citarum rivers analyzed using ICP-AES method.

Reference

1. Google Maps of South East Asia. Available online: https://www.google.com/maps/place/South+East+Asia/@-1.8815737,108.9124474,6z/data=!4m5!3m4!1s0x3233af605e720cd5:0x28a70f18542d1b91!8m2!3d-2.2179704!4d115.66283 (accessed on 6 August 2019).
